# Honeycomb blocks composed of carbonate apatite, β-tricalcium phosphate, and hydroxyapatite for bone regeneration: effects of composition on biological responses

**DOI:** 10.1016/j.mtbio.2019.100031

**Published:** 2019-09-24

**Authors:** K. Hayashi, R. Kishida, A. Tsuchiya, K. Ishikawa

**Affiliations:** Department of Biomaterials, Faculty of Dental Science, Kyushu University, 3-1-1 Maidashi, Higashi-ku, Fukuoka, 812-8582, Japan

**Keywords:** Bone-graft substitute, Scaffold, Bone regeneration, Osteogenesis, Osteogenic differentiation, honeycomb blocks, HCBs, hydroxyapatite, HAp, tricalcium phosphate, TCP, carbonate apatite, CO_3_Ap, calcium phosphate, CaP, osteoclasts, OCs, honeycomb, HC, blood vessels, BV, Fourier transform infrared, FTIR, postoperative week, POW, hematoxylin-eosin, HE, osteoblast, OB, mesenchymal stem cells, MSCs

## Abstract

Synthetic scaffolds exhibiting bone repair ability equal to that of autogenous bone are required in the fields of orthopedics and dentistry. A suitable synthetic bone graft substitute should induce osteogenic differentiation of mesenchymal stem cells, osteogenesis, and angiogenesis. In this study, three types of honeycomb blocks (HCBs), composed of hydroxyapatite (HAp), β-tricalcium phosphate (TCP), and carbonate apatite (CO_3_Ap), were fabricated, and the effects of HCB composition on bone formation and maturation were investigated. The HC structure was selected to promote cell penetration and tissue ingrowth. HAp and β-TCP HCBs were fabricated by extrusion molding followed by sintering. The CO_3_Ap HCBs were fabricated by extrusion molding followed by sintering and dissolution-precipitation reactions. These HCBs had similar macroporous structures: all harbored uniformly distributed macropores (∼160 ​μm) that were regularly arrayed and penetrated the blocks unidirectionally. Moreover, the volumes of macropores were nearly equal (∼0.15 ​cm^3^/g). The compressive strengths of CO_3_Ap, HAp, and β-TCP HCBs were 22.8 ​± ​3.5, 34.2 ​± ​3.3, and 24.4 ​± ​2.4 ​MPa, respectively. Owing to the honeycomb-type macroporous structure, the compressive strengths of these HCBs were higher than those of commercial scaffolds with intricate three-dimensional or unidirectional macroporous structure. Notably, bone maturation was markedly faster in CO_3_Ap HCB grafting than in β-TCP and HAp HCB grafting, and the mature bone area percentages for CO_3_Ap HCBs at postsurgery weeks 4 and 12 were 14.3- and 4.3-fold higher and 7.5- and 1.4-fold higher than those for HAp and β-TCP HCBs, respectively. The differences in bone maturation and formation were probably caused by the disparity in concentrations of calcium ions surrounding the HCBs, which were dictated by the inherent material resorption behavior and mechanism; generally, CO_3_Ap is resorbed only by osteoclastic resorption, HAp is not resorbed, and β-TCP is rapidly dissolved even in the absence of osteoclasts. Besides the composition, the microporous structure of HC struts, inevitably generated during the formation of HCBs of various compositions, may contribute to the differences in bone maturation and formation.

## Highlights

• Honeycomb (HC) blocks, containing hydroxyapatite (HAp), β-tricalcium phosphate (TCP), and carbonate apatite, were fabricated.
• These HC blocks have higher compressive strengths than commercial scaffolds.
• Carbonate apatite HC blocks had higher postsurgery mature bone area percentages than HAp and β-TCP HC blocks.
• Concentration of calcium ions surrounding HC blocks may affect bone maturation and formation.
• HC strut microporous structure may also affect bone maturation and formation.

## 1 Introduction

Autologous bone, which has been recognized as the gold standard material for bone grafting, has many disadvantages as it lengthens the surgical procedure and causes donor site morbidity [1,2]. Numerous complications can occur after harvesting the bone graft from the donor site, which can potentially impair the patient's quality of life [[3], [4], [5], [6], [7], [8]].

In terms of supply, invasiveness, and cost, synthetic materials are preferred as scaffolds [[9], [10], [11], [12], [13]]. Bioactivity, biocompatibility, and osteoconductivity are essential requirements for the development of suitable scaffolds for bone grafting, and they can be tremendously affected by the scaffold's material composition [[9], [10], [11], [12], [13]].

Calcium phosphate (CaP), silicate glass, and composites of silicate glass and minerals or polymers are well-known materials with high bioactivity, biocompatibility, and osteoconductivity [[9], [10], [11], [12], [13], [14], [15], [16], [17], [18], [19], [20], [21], [22]]. Once these materials are degraded, the released ions become supersaturated with respect to biological apatite (often referred to as bone apatite), resulting in the formation of apatite nanocrystals on the surface of the dissolving material [[9], [10], [11], [12], [13]]. As a result, direct and strong bonding of the bone tissues to the material is developed. Therefore, control over the material's resorption mechanism and rate is crucial for bone regeneration.

Natural bone is resorbed by osteoclastic resorption; however, the resorption of most CaP materials is ruled by different mechanisms and behaviors [23]. Even among CaPs, the resorption mechanisms can greatly differ depending on the material's specific composition [23]. Hydroxyapatite [HAp; Ca_10_(PO_4_)_6_(OH)_2_] is a typical bioactive CaP material for bone grafting [[9], [10], [11], [12], [13]]. Although HAp composition is similar to that of natural bone mineral [[9], [10], [11], [12], [13]], it displays poor resorption properties, and, in some cases, it can stay in the bone defect for more than 10 years after implantation [24]. The poor resorption property of HAp causes minor fractures at the interface between the material and the bone or inside the material, resulting in bone deformation years after surgery [24,25]. Furthermore, the remaining HAp within the host's bone is likely to compromise the intrinsic strength of the bone at the callus site [26,27]. β-tricalcium phosphate [TCP; Ca_3_(PO_4_)_2_] is also a representative bioactive CaP material. β-TCP spontaneously dissolves under physiological condition and without osteoclastic resorption [23,28]. Therefore, the resorption speed of β-TCP is faster than that of natural bone mineral, indicating that the rate of β-TCP resorption does not correspond to that of bone formation [29]. The imbalance between resorption and osteogenesis results in the deterioration of the bone quality [30].

Mimicking the composition of natural bone mineral is thought to be an effective way to achieve optimal resorption behavior and to reduce inflammatory responses. Human natural bone mineral is known as AB-type carbonate apatite (CO_3_Ap) where the carbonate ions replace both the hydroxyl and phosphate sites of HAp [31]. The resorption properties of CO_3_Ap are attributed to the tendency of the carbonate to reduce crystallinity within the apatite structure, thereby enhancing bone reformation or turnover [32]. Importantly, CO_3_Ap is resorbed only by osteoclastic resorption, therefore its resorption rate closely matches that of the natural bone [23,33,34]. In previous reports, osteoclastic responses to various CaP materials (CO_3_Ap, HAp, β-TCP, tetracalcium phosphate, α-TCP, dicalcium phosphate dihydrate, and octacalcium phosphate) were studied *in vitro*. Of those materials, only CO_3_Ap was resorbed by osteoclasts (OCs) [23] and it was shown that the morphology of OCs cultured on CO_3_Ap was similar to that of OCs of natural bone [23]. By contrast, the dissolution of β-TCP, tetracalcium phosphate, α-TCP, and dicalcium phosphate dihydrate was not associated with osteoclastic resorption and caused dramatic changes in ion concentration levels in OCs, resulting in the impairment of growth/maturation of OCs [23,35,36].

Upon meeting suitable bioactivity and biocompatibility, the porous structure of the scaffold takes on great significance in bone regeneration as it affects cell penetration, organization, proliferation, differentiation, vascularization, and bone ingrowth. Special attention should be paid to pore size, interconnectivity, permeability, and orientation when designing scaffolds. Generally, the minimum pore size required to regenerate mineralized bone is considered to be 100 ​μm [37]. However, this pore size threshold was obtained in research using three-dimensional porous scaffolds with ununiform macropore size. Bone ingrowth was also studied in scaffolds with relatively uniform macropore size produced by laser perforation techniques. The report shows that scaffolds with different pore sizes (50, 75, 100, and 125 ​μm) promoted bone ingrowth in a similar way [38]. In addition to pore size, interconnectivity and permeability play important roles in tissue regeneration. Interconnected pores are required for oxygen, nutrient, and waste transport, whereas permeability relates to the ease of flow through an interconnected porous structure [39]. Furthermore, permeability depends on not only pore size but also pore shape and orientation: pores that penetrate straight through a material are presumed to enhance permeability to a greater extent than interconnected pores featuring intricate shapes [39].

Macroporous scaffolds have generally been fabricated by first mixing appropriate amounts of transient porogens with powders and then evaporating, burning out, or dissolving the porogen or spacer. However, although the porosity, pore shape, and pore size can be controlled to a certain extent by varying the shape, size, and volume fraction of the porogens, the macroporous structure formed is intricate [15,19,22,[40], [41], [42], [43], [44], [45], [46], [47]]. Furthermore, when porosity is increased by using the aforementioned methods, the mechanical strength of the scaffolds is substantially decreased. Scaffolds with insufficient mechanical properties are unable to resist fatigue and have insufficient load-bearing properties, thereby being susceptible to collapse or internal fracture [48], which limits their application in weight-bearing areas [49,50].

The honeycomb (HC) structure, which is fabricated through extrusion molding by using a HC die-equipped extruder, seems to be a favorable porous structure for cell penetration, vascularization, and bone ingrowth. Its pore size and shape can be accurately controlled through die design: the resulting pores are arranged regularly and penetrate straight through the scaffold in one direction. Localization of cells involved in bone remodeling within a 200-μm range of a blood vessel ​(BV) supply was reported to be essential for cells to access nutrients and remove waste [51]. The uniform macropore size and regular arrangement of the HC structure are considered to be suitable for providing a conductive environment for cell survival and continuous bone remodeling. Furthermore, owing to the ordered macroporous structure, the HC structure is expected to bear greater loads applied in the pore direction than other types of structure.

In this study, we fabricated HC-type porous structure blocks (HC blocks, or HCBs) composed of CO_3_Ap, HAp, and β-TCP and analyzed their structural and mechanical properties. Furthermore, we investigated the influence of material composition on resorption speed, new bone formation, and efficacy of bone repair by means of animal experiments conducted in rabbits.

## 2 Materials and methods

### 2.1 Materials

HAp and β-TCP powders (purity ​≥ ​99.9%) were purchased from Taihei Chemical Industrial Co., Ltd. (Osaka, Japan). Calcium sulfate (CaSO_4_) powder (purity ​≥ ​97%) was purchased from Nacalai Tesque (Kyoto, Japan), and it was heated at 700 ​°C for 6 ​h before use. β-glycerol phosphate (purity ​≥ ​96%), ascorbic acid (purity ​≥ ​99.6%), cell lysis buffer M, *p*-nitrophenyl phosphate (purity ​≥ ​99%), and rapid protein assay kit were purchased from Wako Pure Chemical Industries Ltd. (Osaka, Japan). Povidone-iodine solution was purchased from Meiji Seika Pharma (Tokyo, Japan). Wax-based binder was purchased from Nagamine Manufacturing Co., Ltd. (Kagawa, Japan).

### 2.2 Fabrication of CO_3_Ap HCBs

**Fig. 1:**
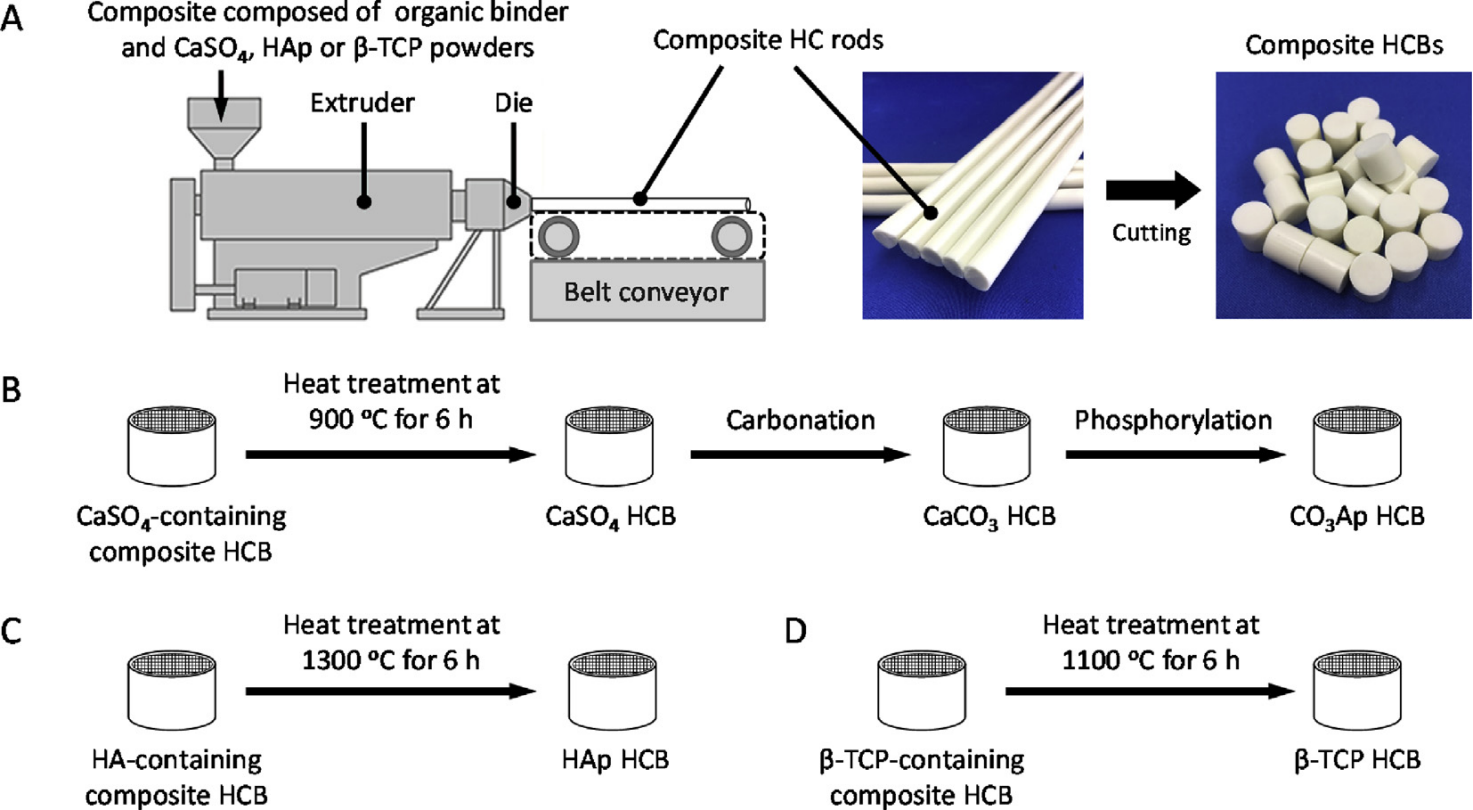
**HCBs fabrication.** (A) Extrusion and fabrication of HCBs composed of a mixture of a wax-based binder and CO_3_Ap, HAp, or β-TCP powder. Fabrication schemes of CO_3_Ap HCBs (B), HAp HCBs (C), and β-TCP HCBs (D).

CaSO_4_ powder was mixed with a wax-based binder at 150 ​°C for 3 ​h by using a Labo Plastomill M roller mixer (Toyo Seiki Co., Nagano, Japan) fitted with a uniaxial extruder. The mixture was extruded at 95 ​°C using a HC extrusion die with 150-μm windows and a 300-μm pitch to generate HC rods. A schematic of the extrusion and HCB fabrication processes is shown in Fig. 1. The HC rods were cut to the desired lengths to fabricate the blocks. HCBs consisting of the binder containing CaSO_4_ were heated at 0.15 ​°C/min to 900 ​°C in a box furnace and kept there for 24 ​h (Nitto Kagaku, Nagoya, Japan) to remove the binder. The CaSO_4_ HCBs were immersed in a mixture of 2 ​M NaHCO_3_ and 2 ​M Na_2_CO_3_ and stored in a VTEC-18 incubator (Isuzu CAP, Niigata, Japan) at 40 ​°C for 4 days to convert its composition to CaCO_3_. The CaCO_3_ HCBs were washed 10 times with water, immersed in a 1 ​M Na_2_HPO_4_ solution, and stored in an incubator at 80 ​°C for 7 days to convert the composition to CO_3_Ap. The CO_3_Ap HCBs were rinsed with water 10 times.

### 2.3 Fabrication of HAp and β-TCP HCBs

HAp or β-TCP powder was mixed with a wax-based binder at 150 ​°C for 3 ​h by using a Labo Plastomill M roller mixer fitted with a uniaxial extruder. The mixture was extruded at 95 ​°C using an HC extrusion die with 150-μm windows and a 300-μm pitch to generate HC rods. A schematic of the extrusion and HCB fabrication processes is shown in Fig. 1. The HC rods were cut to the desired lengths to fabricate the blocks. HCBs consisting of the binder containing HAp or β-TCP were heated at 0.15 ​°C/min to 1300 or 1100 ​°C, respectively, in a box furnace and kept there for 24 ​h to remove the binder and to sinter.

### 2.4 Characterization of HCBs

HCB compositions were determined using powder X-ray diffraction (XRD) analysis. Samples were pulverized, and the XRD patterns were recorded on a D8 Advance diffractometer (Bruker AXS GmbH, Karlsruhe, Germany) using Cu Kα radiation at 40 ​kV and 40 ​mA. The samples were scanned in continuous mode over a diffraction range of 20°–60° (2θ).

Fourier transform infrared (FTIR) spectra were measured with a spectrometer (FT/IR-6200; JASCO, Tokyo, Japan) by using the KBr disc method.

HC structures were confirmed using stereomicroscopy and scanning electron microscopy (SEM; S3400 ​N, Hitachi High Technologies, Tokyo, Japan). The accelerating voltage was 15 ​kV. Before analysis, samples were coated with Au–Pd by using an MSP-1S magnetron sputtering source (Vacuum Device Co., Ibaraki, Japan).

The carbonate content of samples was measured through elemental analysis or using a CHN coder (MT-6; Yanako Analytical Instruments, Kyoto, Japan).

Pore-size distribution was determined using a mercury porosimeter (AutoPore 9420, Shimadzu, Kyoto, Japan).

The mechanical strengths of samples were evaluated in terms of compressive strength. The diameter of each sample was measured using a digital micrometer (IP65, Mitutoyo, Kanagawa, Japan), and the samples were then vertically compressed by using a load cell at a crosshead speed of 1 ​mm ​min^−1^ in a universal testing machine (Autograph AGS-J, Shimadzu, Kyoto, Japan). The compressive strength values reported are the average of eight samples.

### 2.5 *In vitro* evaluation of cell attachment to HCBs and alkaline phosphatase ​activity

MC3T3-E1 cells were seeded onto samples in 24-well plates at an initial density of 6 ​× ​10^4^ ​cells per well and cultured in differentiation medium consisting of normal culture medium supplemented with 10 ​mM β-glycerol phosphate and 50 ​μg ​mL^−1^ ascorbic acid. After 7 days, the cells were washed twice with PBS and lysed with cell lysis buffer M containing 20 ​nM Tris-HCl, 200 ​nM sodium chloride, 2.5 ​mM magnesium chloride, and 0.05% NP-40 at room temperature for 30 ​min. After centrifugation, 20 ​μL of the lysate was incubated with 6.7 ​mM *p*-nitrophenyl phosphate at 37 ​°C for 15 ​min, the optical density was measured at 405 ​nm, and the alkaline phosphatase (ALP) activity was calculated by interpolation from a standard curve. The relative ALP activity was normalized against the total protein concentration measured using a rapid protein assay kit (Wako Pure Chemical Industries Ltd.). Then, 10 ​μL of the cell-lysis solution was pipetted into a microdisc well containing 250 ​μL of the chromogen solution, and after incubation for 30 ​min ​at 37 ​°C, the 600-nm absorbance was measured in a disc reader (n ​= ​4 per group).

### 2.6 *In vivo* evaluation of HCB replacement with bone

The protocols used for the animal experiments were approved by the Animal Care and Use Committee of Kyushu University (no. A30–338-0; issued December 19, 2018). Four 3.0- to 3.5-kg male Japanese white rabbits aged 18–19 weeks old (obtained from Japan SLC, Hamamatsu, Japan) were included in each experimental group. The animals were housed in the animal center of the University and maintained on a standard diet and water. The rabbits were anesthetized with an intraperitoneal injection of a mixture of ketamine (30 ​mg ​kg^−1^) and xylazine (5.0 ​mg ​kg^−1^). The rear limbs were shaved to remove the fur, and the skin was disinfected with 10% povidone-iodine solution. The distal epiphysis of the femur was used to ensure adequate bone volume. A 6-mm hole was drilled into the femur of each animal and the CO_3_Ap, HAp, and β-TCP HCBs were poured into the holes. The fasciae were then sutured shut. The HCBs were implanted bilaterally in each animal. The rabbits were allowed unrestrained movement in their cages after recovering from anesthesia. At postoperative week (POW) 4 and 12, the rabbits were euthanized in batches and the distal epiphysis of each femur was harvested (n ​= ​4 per group). The resorption and bone formation properties of the HCBs were evaluated by means of microcomputed tomographic (μ-CT) scanning and histological analysis. The area% values of materials and mineralized bone in each defect were estimated from hematoxylin-eosin-stained tissue sections by using a BZ-X digital analyzer (Keyence, Osaka, Japan).

### 2.7 Statistical analysis

Student's *t*-test was used to compare group means. *p* ​< ​0.05 was considered statistically significant.

## 3 Results and discussion

### 3.1 Fabrication and characterization of HCBs composed of CO_3_Ap, HAp, and β-TCP

**Fig. 2:**
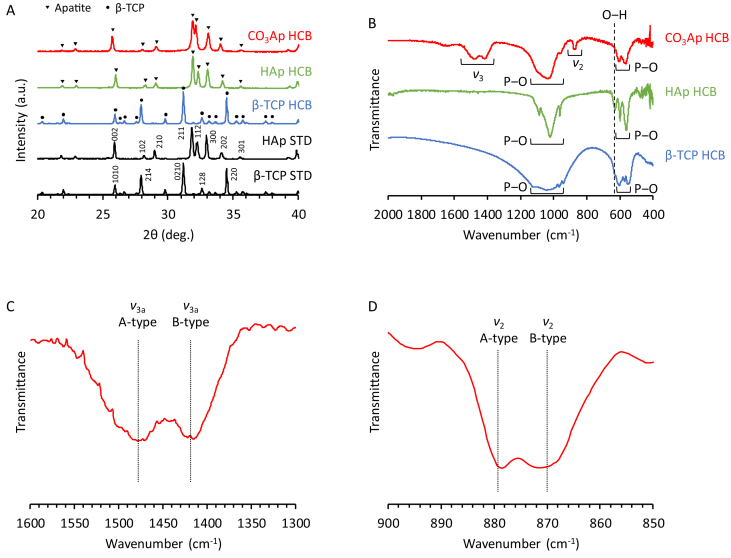
**Characterization of HCBs.** (A) XRD patterns of CO_3_Ap HCBs, HAp HCBs, β-TCP HCBs, HAp standard substance (STD), and β-TCP STD. (B) FTIR spectra of CO_3_Ap, HAp, and β-TCP HCBs. (C) FTIR spectra of CO_3_Ap HCBs in the 1600‒1300 ​cm^−1^ carbonate *ν*_3_ region. (D) FTIR spectra of CO_3_Ap HCBs in the 900‒850 ​cm^−1^ carbonate *ν*_2_ region.

HCBs composed of CO_3_Ap, HAp, and β-TCP were fabricated as follows (Fig. 1): (1) Three types of composites composed of organic binder and CaSO_4_, HAp, or β-TCP were prepared. (2) The CaSO_4_-, HAp-, or β-TCP-containing composites were extruded through the HC extrusion die to generate the corresponding composite HC rods. (3) The CaSO_4_-, HAp-, or β-TCP-containing composite HC rods were cut at regular intervals into the corresponding composite HCBs of uniform length. (4) The CaSO_4_-, HAp-, and β-TCP-containing composite HCBs were subjected to heat treatment at 900, 1300, and 1100 ​°C, respectively, to remove organic components and sinter the corresponding ceramic powders (Fig. 1B‒D). As a result, CaSO_4_, HAp, and β-TCP HCBs were obtained. The XRD patterns confirmed that HAp and β-TCP HCBs were composed of single-phase HAp and β-TCP, respectively (Fig. 2A). (5) The composition of the CaSO_4_ HCBs was converted to CaCO_3_ by first dissolution-precipitation reactions, i.e., carbonation reactions, conducted by immersing the CaSO_4_ HCBs in a solution containing Na_2_CO_3_ and NaHCO_3_ (Fig. 1B). (6) The composition of the CaCO_3_ HCBs was converted to CO_3_Ap by dissolution-precipitation reactions, i.e., phosphorylation reactions, by immersing the CaCO_3_ HCBs in a Na_2_HPO_4_ solution. The XRD patterns showed that the diffraction patterns of CO_3_Ap HCBs coincided with that of the HAp standard, indicating that an apatite phase was formed (Fig. 2A). Furthermore, the apatite lattice constants along the *a*-axis and *c*-axis for CO_3_Ap HCBs, estimated from (002) and (100) reflection planes, were 12.186 and 6.916 ​Å, respectively. The *a*- and *c*-lattice constants for HAp are 9.423 and 6.889, respectively [52]. Thus, both *a*- and *c*-lattice constants for CO_3_Ap HCBs were larger than those for HAp. It was reported that both *a*- and *c*-lattice constants of AB-type CO_3_Ap are larger than those of HAp, whereas the *c*-lattice constants of A-type CO_3_Ap and the *a*-axis of B-type CO_3_Ap are smaller than those of HAp [52]. These results demonstrated that CO_3_Ap HCBs were composed of AB-type CO_3_Ap, not of A- and B-type CO_3_Ap.

The foregoing heat-treatment temperature was determined based on the following considerations: (1) the material composition varies with heat-treatment temperature; (2) the speed of dissolution-precipitation reactions depends on the degree of sintering (i.e., heat-treatment temperature) only in the case of CO_3_Ap; and (3) the mechanical properties depend on the degree of sintering. Therefore, to obtain the highest mechanical strength possible, we selected the maximum temperature necessary to obtain the target composition. In detail, for the fabrication of CO_3_Ap HCBs, heat treatment was conducted at 900 ​°C to obtain CaSO_4_ HCBs; this temperature was selected for the following reasons: CaO began to form when CaSO_4_ HCBs were heated at temperatures above 900 ​°C. CaO was the cause of crack formation in the HCBs because considerable volume change occurred when CaO reacted with water vapor in the atmosphere to generate Ca(OH)_2_. Furthermore, as the degree of sintering increased, the speed of dissolution-precipitation reactions (especially, the second dissolution-precipitation reactions, i.e., phosphorylation reaction) decreased because the HC struts became denser due to micropore decrease. Consequently, the reaction solution hardly penetrated into the strut center. Therefore, to complete the composition conversion from CaSO_4_ to CO_3_Ap within an acceptable manufacturing period, the heat-treatment temperature was restricted to ≤900 ​°C. In the fabrication of β-TCP HCBs, the heat-treatment temperature was limited to a maximum of ∼1100 ​°C because the phase transition of β-TCP to α-TCP occurs at 1150 ​± ​30 ​°C [53]. By contrast, HAp HCBs were sintered at 1300 ​°C because HAp is stable at this temperature and α-TCP formation begins at 1475 ​°C [54]. Thus, each HCB was subject to heat treatment at a specific temperature, and this influenced the microporosity and mechanical strength of the distinct HCBs, as described later.

In the FTIR spectra of CO_3_Ap, HAp, and β-TCP HCBs, the phosphate absorption bands appeared at 540–605 and 940‒1130 ​cm^−1^ (Fig. 2B) [55]. In the HAp HCB spectrum, the absorption band attributed to hydroxyl was observed at 628 ​cm^−1^ [55]. By contrast, in the spectrum of CO_3_Ap HCBs, the hydroxyl band was absent and the bands corresponding to carbonate appeared at 1350‒1550 ​cm^−1^ (carbonate *ν*_3_ region) and 855‒890 ​cm^−1^ (carbonate *ν*_2_ region) [52,55]. The FTIR spectra of CO_3_Ap HCBs in the carbonate *ν*_3_ region showed that A- and B-type *ν*_3_ vibrations were found at 1478 and 1415 ​cm^−1^, respectively (Fig. 2C). The FTIR spectra of CO_3_Ap HCBs in the carbonate *ν*_2_ region showed that A- and B-type *ν*_2_ vibrations were found at 878 and 872 ​cm^−1^, respectively (Fig. 2D). Thus, the FTIR and XRD results demonstrated that CO_3_Ap HCBs were composed of AB-type CO_3_Ap.

The results of CHN elemental analysis showed that the carbonate content of CO_3_Ap HCBs was 12.1 ​± ​2.4%. Human hard tissues contain 11 ​± ​1% of carbonate ions [56]. Thus, carbonate content of CO_3_Ap HCBs is nearly equal to that of human hard tissues.

**Fig. 3:**
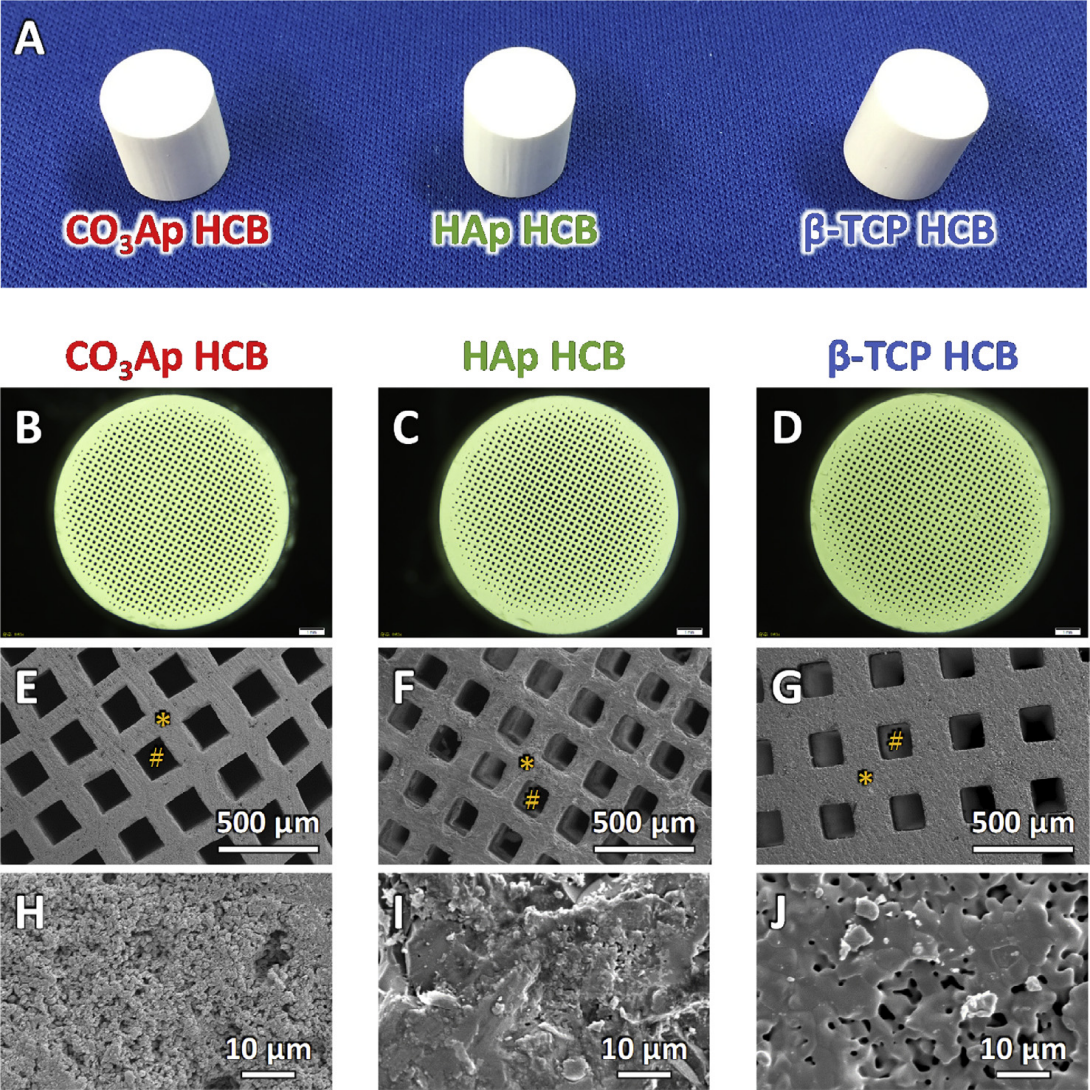
**Examination of HCB structures.** Photograph of CO_3_Ap, HAp, and β-TCP HCBs (A). Stereomicroscopy images of CO_3_Ap (B), HAp (C), and β-TCP HCBs (D). SEM images of CO_3_Ap (E, H), HAp (F, I), and β-TCP HCBs (G, J). “*” and “#” indicate HC strut and macropore, respectively. Magnified SEM images of the struts of CO_3_Ap (H), HAp (I), and β-TCP HCBs (J).

All CO_3_Ap, HAp, and β-TCP HCBs were white-columned blocks harboring regularly arranged square through-macropores (Fig. 3A‒C). The macropore sizes of CO_3_Ap, HAp, and β-TCP HCBs were uniform: 161 ​± ​5, 153 ​± ​2, and 166 ​± ​2 ​μm, respectively (Fig. 3C). The struts of CO_3_Ap and β-TCP HCBs contained micropores, i.e., ≤ 10 ​μm pores [57], whereas the struts of HAp HCB were dense and lacked micropores (Fig. 3D). Furthermore, the crystals in the struts of CO_3_Ap HCBs were isolated from one another, whereas adjacent crystals were incorporated in β-TCP HCBs by sintering (Fig. 3D); consequently, the micropores in the struts of CO_3_Ap HCBs were markedly smaller than those in the struts of β-TCP HCBs (Fig. 3D).

**Fig. 4:**
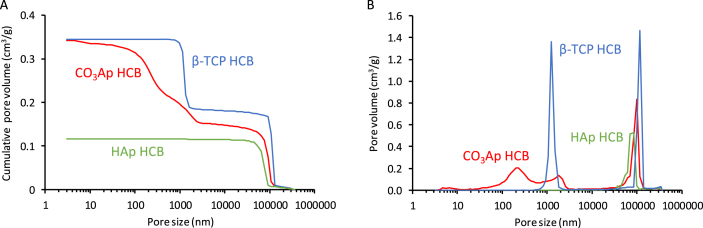
**Characterization of HCBs' porosity.** (A) Cumulative pore volume and (B) pore-size distribution vs. pore diameter of CO_3_Ap, HAp, and β-TCP HCBs measured using the mercury intrusion method.

The pore-size distributions and total porosities of CO_3_Ap, HAp, and β-TCP HCBs were measured using mercury intrusion (Fig. 4). For CO_3_Ap, HAp, and β-TCP HCBs, the macropore volumes measured were, respectively, 0.15, 0.11, and 0.18 ​cm^3^ ​g^−1^ (Fig. 4A) and the micropore volumes were 0.20, 0.00, and 0.16 ​cm^3^ ​g^−1^, respectively. The total porosities for CO_3_Ap, HAp, and β-TCP HCBs were, respectively, 45.7, 26.9, and 49.3%. Thus, CO_3_Ap and β-TCP HCBs showed small differences in macropore and micropore volumes and total porosity. However, the size distributions of the micropores were different between these two HCBs (Fig. 4B): the micropores in CO_3_Ap HCBs covered a broad size range, from tens of nanometers to a few micrometers, whereas the micropores in β-TCP HCBs were uniformly distributed in size (∼1–2 ​μm). Malina et al. reported that the density and the shrinkage percentage of HAp were dramatically increased, while its porosity was dramatically decreased when using temperatures ranging from 800 ​°C to 1100 ​°C [58]. Above 1100 ​°C, its density, shrinkage percentage, and porosity did not change significantly [58]. Thus, HAp is almost completely sintered at 1300 ​°C, which corresponds to the sintering temperature we used to fabricate HAp HCBs. These findings explain the absence of micropores in HAp HCBs. For β-TCP, Wong et al. reported that the total porosity and micropores remained unchanged at temperatures from 1100 ​°C to 1200 ​°C, while being dramatically decreased from 1200 ​°C to 1300 ​°C [59]. Thus, β-TCP is not completely sintered at 1100 ​°C, which corresponds to the sintering temperature for fabricating β-TCP HCBs. Therefore, micropores remained in β-TCP HCBs.

### 3.2 Mechanical properties

**Fig. 5:**
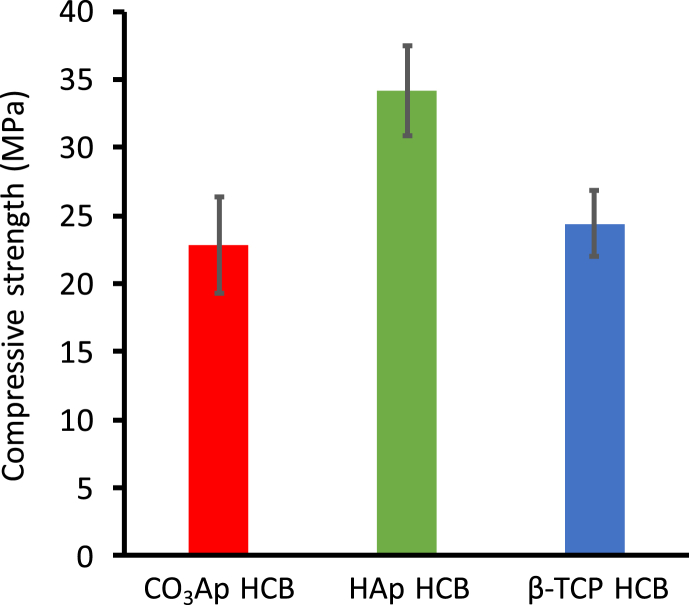
**HCB compressive strengths.** Compressive strengths measured for CO_3_Ap, HAp, and β-TCP HCBs.

The compressive strengths of CO_3_Ap, HAp, and β-TCP HCBs were 22.8 ​± ​3.5, 34.2 ​± ​3.3, and 24.4 ​± ​2.4 ​MPa, respectively (Fig. 5). Thus, the compressive strength of CO_3_Ap HCBs was nearly equal to that of β-TCP HCBs, and HAp HCBs exhibited higher compressive strength than CO_3_Ap and β-TCP HCBs. These results were expected because compressive strength typically increases with decreasing porosity.

As compared with commercial synthetic scaffolds, CO_3_Ap, HAp, and β-TCP HCBs exhibited substantially higher compressive strengths. For example, the compressive strengths of three scaffolds harboring irregular macropores, Neobone®, Apaceram-AX®, and Superpore®, are 8–10, 2, and 5 ​MPa, respectively [28]. Furthermore, the HAp scaffold with unidirectional macroporous structure is marketed as Regenos® [50] and its macroporous structure resembles frost columns rather than honeycomb. The compressive strength of Regenos® along the macropores is 14 ​MPa [50], which is considerably lower than the compressive strengths of the HCBs: the straight struts present in HCBs are separate, whereas the struts in Regenos® are not completely straight and lean against each other. Orientation, linearity, and independence are all critical for increasing compressive strength along the macropores while maintaining high porosity. Therefore, the HC structure seems to be an optimal structure as it possesses both high mechanical strength and high porosity. Moreover, it is reported that Regenos® is not suitable for open-door laminoplasty because of its insufficient mechanical strength [50], while HCBs could be potentially used in this procedure owing to their high mechanical strength.

### 3.3 *In vitro* studies

**Fig. 6:**
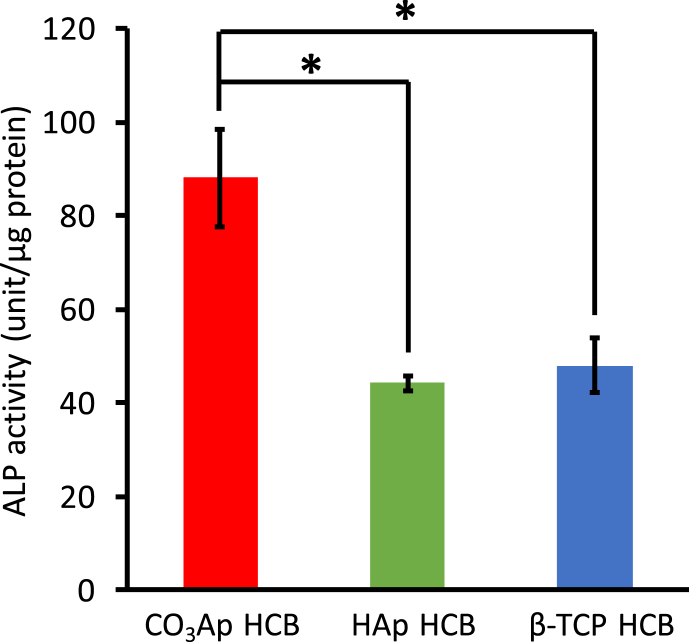
**HCB effect on MC3T3-E1 cell differentiation *in vitro*.** ALP activity of cells cultured for 7 days on CO_3_Ap, HAp, and β-TCP HCBs; *p ​< ​0.05.

MC3T3-E1 cell differentiation was quantified by measuring ALP activity as a marker for osteoblast (OB) maturation. ALP activity of MC3T3-E1 cells incubated on CO_3_Ap HCBs was roughly twofold higher than that of MC3T3-E1 cells incubated on β-TCP and HAp HCBs (Fig. 6).

### 3.4 *In vivo* studies

**Fig. 7:**
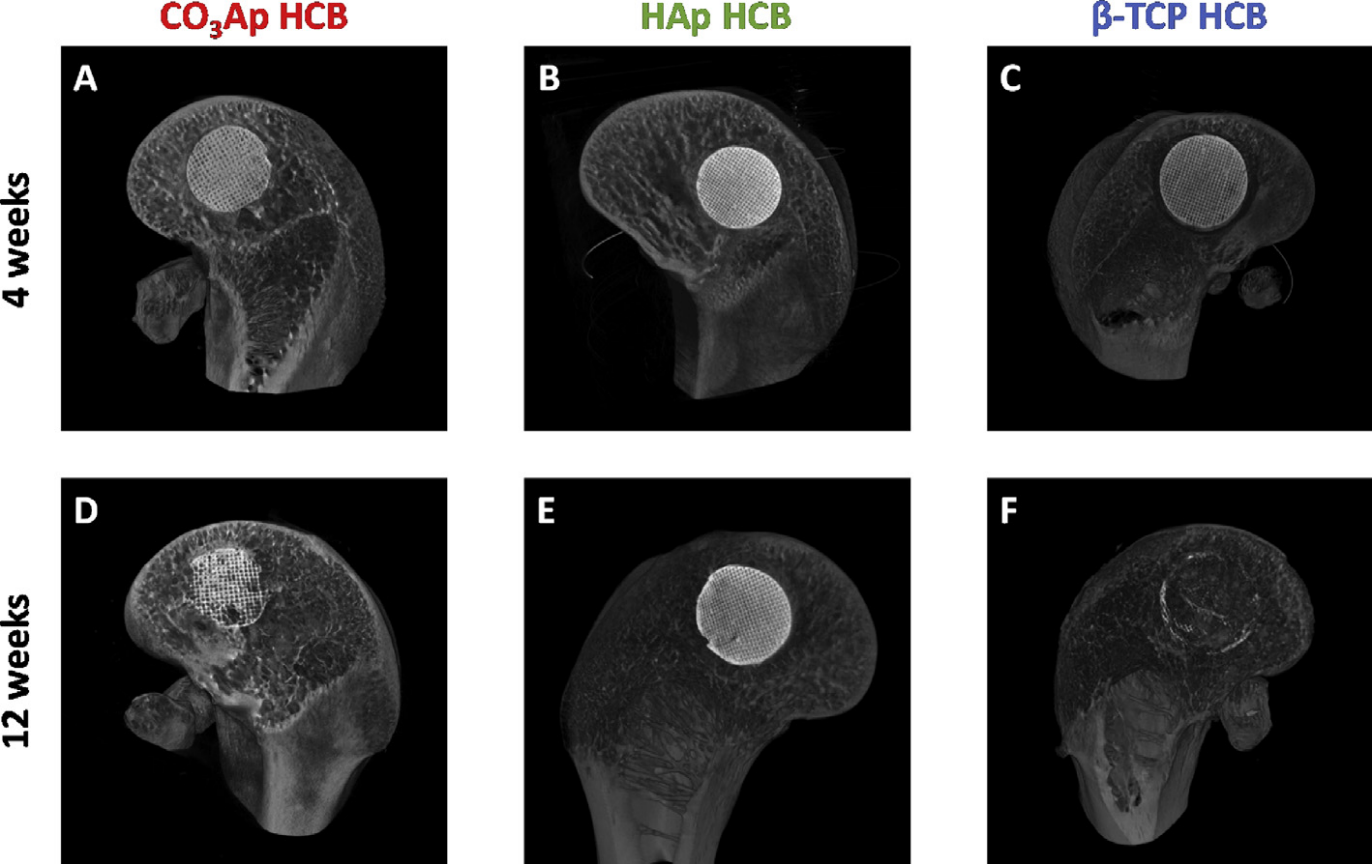
**Examination of HCB resorption *in vivo*.** μ-CT images of bone defects at POW 4 and 12 for CO_3_Ap (A, D), HAp (B, E), and β-TCP (C, F) HCBs, respectively.

CO_3_Ap, HAp, and β-TCP HCBs were implanted into defects drilled in the distal epiphysis of rabbit femurs. X-ray μ-CT analysis revealed that, at POW 4, only modest resorption of CO_3_Ap, HAp, and β-TCP HCBs occurred and that the HC structure remained intact in all samples (Fig. 7A‒C). At POW 12, partial resorption of CO_3_Ap HCBs was visible, although the implanted HCBs remained in place and retained the HC structure (Fig. 7D). Insignificant to no resorption of HAp HCBs was observed (Fig. 7E), whereas in the case of β-TCP HCBs, large areas were resorbed, and only small amounts of the material were found (Fig. 7F).

**Fig. 8:**
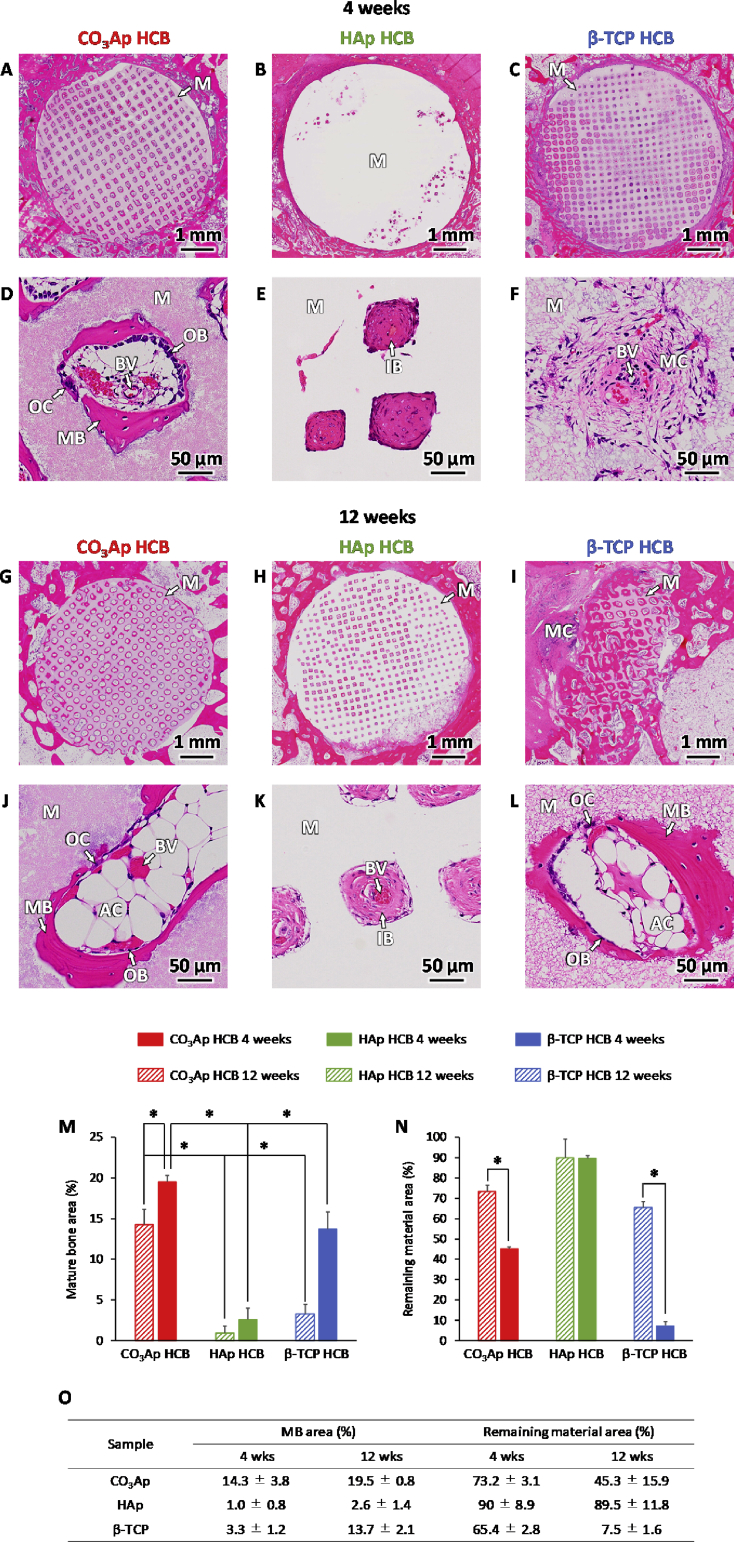
**Histological analysis of HCB resorption *in vivo*.** Hematoxylin-eosin-stained histological images of bone defects at POW 4 for CO_3_Ap (A, D), HAp (B, E), and β-TCP (C, F) HCBs and at POW 12 for CO_3_Ap (G, J), HAp (H, K), and β-TCP (I, L) HCBs. “OB”, “OC”, “MB”, “IB”, “MC”, “M”, “BV”, and “AC” indicate osteoblast, osteoclast, new mature bone, immature bone, mesenchyme, material, blood vessel, and adipose cell, respectively. Area% of mature bone (M) and remaining material (N) at POW 4 and 12 for CO_3_Ap, HAp, and β-TCP HCBs; *p ​< ​0.01. Table showing the area% of mature bone and remaining material at POW 4 and 12 for CO_3_Ap, HAp, and β-TCP HCBs (O).

Histological images of femurs implanted with CO_3_Ap HCBs showed that, at POW 4, new mature bone (MB) was formed along the walls surrounding the macropores, OBs were present along the new bone, osteoclasts (OCs) appeared on the material surface, and BVs ​were formed in the macropores (Fig. 8A, D). The macropores of CO_3_Ap HCBs were deformed, which demonstrated that the HCB material could be resorbed by the OCs. At POW 12 (Fig. 8G, J), the macropores of CO_3_Ap HCBs were further deformed and coalesced with adjacent macropores. However, the material was still in place, the HC structure was maintained, and adequate resorption was achieved. Similar to the observations made on POW 4, OBs and OCs were present in the macropores and MB was formed along the walls surrounding the macropores. Thus, bone remodeling within the macropores was constant over the 12-week period, which resulted in gradual replacement of CO_3_Ap HCBs by new MB. In HAp HCBs, immature bone (IB) was observed in only a small portion of the macropores at POW 4 and no MB was detected (Fig. 8B, E). OBs, OCs, and BVs were not observed in any of the macropores of HAp HCB, whereas these cells and tissues were present in every macropore examined in CO_3_Ap HCBs. At POW 12, almost all macropores were occupied by IB but neither OBs nor OCs were observed in the macropores (Fig. 8H, K). Regarding β-TCP HCBs at POW 4, mesenchyme was present in almost all macropores but OBs and OCs were not detected in the macropores (Fig. 8C, F); new MB formation was observed within a small portion of macropores. By POW 12, a large part of the β-TCP HCBs had been resorbed (Fig. 8I, L). MB was formed surrounding the remaining material, and OBs and OCs were present on the new MB surface and the material surface, respectively.

The percentages of newly formed MB area and remaining material area were estimated from the histological images, and the following results were obtained for CO_3_Ap, HAp, and β-TCP HCBs (respectively, in %): MB area: 14.3 ​± ​3.8, 1.0 ​± ​0.8, and 3.3 ​± ​1.2 ​at POW 4, and 19.5 ​± ​0.8, 2.6 ​± ​1.4, and 13.7 ​± ​2.1 ​at POW 12 (Fig. 8M and Table 8O); remaining material area: 73.2 ​± ​3.1, 90 ​± ​8.9, and 65.4 ​± ​2.8 ​at POW 4, and 45.3 ​± ​15.9, 89.5 ​± ​11.8, 7.5 ​± ​1.6 ​at POW 12 (Fig. 8N and Table 8O). Thus, the MB area percentage in CO_3_Ap HCBs was 14.3- and 4.3-fold higher than the percentages in HAp and β-TCP HCBs at POW 4. At POW 12, the MB area percentage in CO_3_Ap HCBs was 7.5- and 1.4-fold higher than those in HAp and β-TCP HCBs. The aforementioned *in vivo* results demonstrate that HCB composition strongly influenced the material resorption rate, the speed and degree of bone maturation, and the amount and rate of new bone formation.

Regarding HAp resorption, Ogose et al. reported that HAp is not resorbed and is entirely retained in the human bone defect for periods longer than 130 months after implantation [24]. In the present study, we show that HAp HCBs were practically intact by POW 12 (i.e., insignificant to no resorption occurred), which is in line with the previous report. By contrast, β-TCP spontaneously dissolves under physiological condition and in the absence of osteoclastic resorption [23,28] and our *in vivo* results are congruent with these observations: β-TCP HCBs were resorbed extremely rapidly and only a small amount of these HCBs was found at POW 12. It is known that CO_3_Ap is resorbed only by osteoclastic resorption and that its resorption speed and degree are similar to those of natural bone mineral [23,33,34]. We observed that the resorption of CO_3_Ap HCBs notably progressed in the material areas where OCs adhered to and that CO_3_Ap HCBs were not overly rapid resorbed at POW 12.

The aforementioned differences in material resorption speed between CO_3_Ap, HAp, and β-TCP HCBs indicate that these HCBs exhibit different calcium ion release profiles. It is well known that calcium ions are necessary for the development of a functional osteoblast phenotype from primary sources, from mesenchymal-derived stem cells and bone marrow stromal cells [[60], [61], [62], [63]]. Nevertheless, calcium ion concentration levels that are higher than those resulting from natural bone resorption inhibit OC differentiation and osteoclastic resorption [23]. HAp HCB scaffolds did not show significant resorption, and therefore, the levels of calcium ions released were suboptimal, resulting in poor maturation and formation of bone in HAp HCB grafts. On the other hand, the excessively rapid dissolution of β-TCP HCBs resulted in a dramatic increase of calcium ion levels. An excessive local concentration of calcium ions potentially cause biologically adverse effects such as inflammatory response, cell death, or cellular dysfunction as a result of activation of immune response or distortion of the metabolism of cellular calcium [23,35,36,[64], [65], [66]]. In β-TCP HCBs, therefore, the OC differentiation and the osteoclast-driven resorption were inhibited [23,35,36,[64], [65], [66]].

Furthermore, the speed of material resorption can be seen as a scaffold performance indicator as it will largely determine ​the degree of cell attachment and subsequent cell–cell interactions necessary for bone formation and maturation. Rapid resorption of materials causes the disappearance of the scaffold before bone formation and maturation. In this view, β-TCP HCBs underperformed as scaffolds because they were rapidly resorbed in the period between POW 4 and POW 12 before bone was completely formed. The impact of the resorption properties of β-TCP HCBs was also visible in their MB area percentage, which was notably lower than that of CO_3_Ap HCBs. By contrast, maturation and formation of bone are not expected when no scaffold is resorbed because the materials are not involved in bone remodeling [64]. HAp HCBs were not resorbed at POW 12, which resulted in poor bone maturation and formation. This further indicates that HAp HCBs are not involved in bone remodeling, as previously described [67].

The attachment of OBs and OCs and the MB formation along the CO_3_Ap HCB struts occurred during the first four weeks after surgery; the resorption of the material continued gradually until POW 12 and it was accompanied by further cells' attachment and MB formation. Thus, CO_3_Ap HCBs displayed ideal release rates of calcium ion and served adequately as scaffolds. These desirable outcomes are probably owed to the CO_3_Ap-specific resorption behavior through osteoclastic resorption only [68], and thus, CO_3_Ap resorption keeps pace with new bone formation.

In addition to pure composition effects, the effects caused by differences in microporous structures, which are inevitably developed for fabricating CO_3_Ap, HAp, and β-TCP HCBs with sufficient mechanical properties, should be considered. The differences in macroporous structures between these three types of HCBs are considered negligible because the differences in macropore size and volume were only ∼10 ​μm and ∼0.03 ​cm^3^ ​g^−1^, respectively. It has been suggested that the presence of micropores in scaffolds increases the surface area, thereby increasing (1) protein adsorption on the scaffolds [[69], [70], [71], [72]], (2) ion concentration levels around the scaffolds [73], and (3) surface roughness of scaffolds [[74], [75], [76], [77]], which may promote osteogenic differentiation of mesenchymal stem cells (MSCs). Regarding HAp HCBs, no micropores were detected in their struts. The lack of micropores may be one of the reasons for poor maturation and formation of bone in HAp HCB scaffolds. Between CO_3_Ap and β-TCP HCBs, there were small differences in micropore volume and total porosity. However, the size distributions of micropores varied greatly between these two HCBs: the micropores in CO_3_Ap HCBs covered a broad size range, from tens of nanometers to a few micrometers, whereas the micropores in β-TCP HCBs were uniformly distributed in size (∼1–2 ​μm). The differences in micropore size distribution generate the differences in surface roughness and specific surface area. The specific surface areas of CO_3_Ap and β-TCP HCBs were measured by mercury intrusion technique and were 8.65 and 0.531 ​m^2^/g, respectively. CO_3_Ap HCBs have a ∼16-fold higher specific surface area than β-TCP HCBs. Therefore, the differences in the micropore size distribution contributed to the differences in bone maturation and formation *in vivo*, i.e., compared to β-TCP HCB grafting, CO_3_Ap HCB grafting was markedly superior.

Several studies established a comparison between CO_3_Ap HCBs and blocks exhibiting intricate three-dimensional (3D) macroporous structure [78] and between CO_3_Ap HCBs and Regenos®, a commercial scaffold harboring unidirectional macroporous structures [50]. A previous study reported that the edges—but not the centers—of CO_3_Ap blocks exhibiting intricate 3D macroporous structure are resorbed at POW 12 because this type of structure does not allow OC penetration in the blocks [78]. Consequently, no new bone is formed in the central regions of blocks exhibiting intricate macroporous structure [78]. Moreover, in Regenos®, cells cannot reach the central regions of the blocks, which impairs new bone formation there. This is caused by Regenos® macroporous structure, which is similar to that of frost columns with struts that lean against each other and contains imperfect through pores that are heterogeneous in size [79]. By contrast, HC macroporosity facilitates cell distribution throughout the blocks. Thus, HCBs achieve uniform and efficient repair throughout the bone defect, whereas blocks exhibiting a Regenos-like unidirectional and intricate 3D macroporosity can only gradually rebuild the central parts of the bone defects.

In summary, HCBs composition and composition-specific resorption behavior significantly affect bone formation and maturation. Nevertheless, microporous structure, which is inevitably formed for fabricating HCBs with adequate mechanical properties, may be involved in bone formation and maturation. In this study, the microporous structure of the three types of HCBs cannot be fully equalized because the fabrication methods differed substantially between the three compositions, especially between CO_3_Ap HCBs and HAp/β-TCP HCBs: CO_3_Ap was fabricated by sintering followed by dissolution-precipitation, while HAp and β-TCP were fabricated only by sintering. Investigating the effects of microporous structures will contribute to a deeper understanding of their impact on bone formation and maturation. Therefore, in future research, we will clarify the effects of microporous structures on osteogenic differentiation of MSCs and osteogenesis. In addition, we will explore the advantages offered by CO_3_Ap HCBs and investigate their applicability on the treatment of several diseases and traumas.

## 4 Conclusion

HAp, β-TCP, and CO_3_Ap HCBs were successfully fabricated in this study. These HCBs exhibited higher compressive strength than commercial CaP scaffolds harboring irregular macropores and unidirectional macropores. Bone maturation was faster in CO_3_Ap HCBs than in HAp and β-TCP HCBs, and the amount of bone formation was higher in CO_3_Ap HCBs than in HAp and β-TCP HCBs. This is probably a result of the adequate release of calcium ions from CO_3_Ap HCBs during resorption, which makes them suitable as scaffolds. These positive effects are ascribable to (1) the CO_3_Ap-specific resorption behavior and mechanism, i.e., osteoclast-driven resorption ​and (2) the CO_3_Ap resorption rate, which accompanies the formation of new bone. Inferior bone maturation and formation in HAp and β-TCP HCB grafting seem to be caused by no resorption of HAp HCBs and overly rapid dissolution of β-TCP HCBs.

## Conflicts of interest

There are no conflicts of interest to declare in the present study.
